# A Soft-Error-Tolerant SAR ADC with Dual-Capacitor Sample-and-Hold Control for Sensor Systems

**DOI:** 10.3390/s21144768

**Published:** 2021-07-13

**Authors:** Duckhoon Ro, Minseong Um, Hyung-Min Lee

**Affiliations:** 1 School of Electrical Engineering, Korea University, Seoul 02841, Korea; roduckhoon@korea.ac.kr (D.R.); umminsung01@korea.ac.kr (M.U.); 2 Foundry Business, Samsung Electronics, Hwaseong 18448, Korea

**Keywords:** soft-error, radiation-hardened, SAR ADC, sample-and-hold, single event effect, total ionizing dose, sensor system

## Abstract

For a reliable and stable sensor system, it is essential to precisely measure various sensor signals, such as electromagnetic field, pressure, and temperature. The measured analog signal is converted into digital bits through the sensor readout system. However, in extreme radiation environments, such as in space, during flights, and in nuclear fusion reactors, the performance of the analog-to-digital converter (ADC) constituting the sensor readout system can be degraded due to soft errors caused by radiation effects, leading to system malfunction. This paper proposes a soft-error-tolerant successive-approximation-register (SAR) ADC using dual-capacitor sample-and-hold (S/H) control, which has robust characteristics against total ionizing dose (TID) and single event effects (SEE). The proposed ADC was fabricated using 65-nm CMOS process, and its soft-error-tolerant performance was measured in radiation environments. Additionally, the proposed circuit techniques were verified by utilizing a radiation simulator CAD tool.

## 1. Introduction

**Figure 1:**
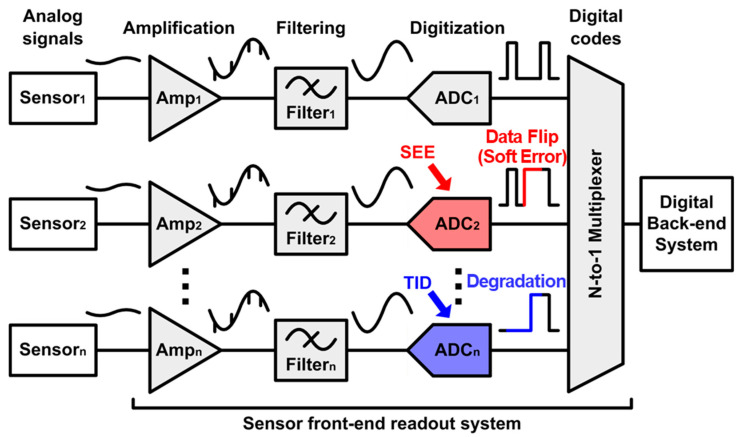
Block diagram of the sensor readout system with radiation effects [8].

For reliable and accurate control of sensor systems, various kinds of analog signals, such as electromagnetic field, pressure, and temperature, should be measured with sensors. The measured analog signal is converted into digital bits through the sensor readout system and transmitted to the digital back-end system as shown in Figure 1. The sensor readout system consists of an amplifier that amplifies the measured analog signal, a filter that removes unnecessary noise or interference, and an analog-to-digital converter (ADC) that converts analog signals into digital bits. In case the performance of the sensor readout system is degraded due to external interferences, such as electromagnetic field, temperature, and radiation effects, the measured signals cannot be accurately transmitted to the digital back-end system, and the sensor system cannot be controlled stably and accurately. Among various types of external interferences degrading the circuit performance, the radiation effects have become a huge problem.

Among the circuits that compose the sensor readout system, the ADC performance is directly related to the stable and accurate control of the system because it determines how accurate the measured analog signal can be converted into digital bits for further data processing. However, the ADCs composed of various types of sub-block circuits can be significantly degraded in harsh radiation environments, such as in space, during flights, and in nuclear fusion reactors, which becomes a major problem in controlling the system [1]. In recent years, the systems that require high reliability, such as autonomous vehicles and optical interconnects, have also been shown to suffer from degradation of circuit performance due to radiation effects [2,3].

Performance degradation of transistors in a complementary metal-oxide-semiconductor (CMOS) integrated circuit (IC) due to high energy radiation, such as alpha particles, neutrons, and gamma rays, can be classified into the total ionizing dose (TID) and the single event effect (SEE) [4,5,6,7]. TID refers to the effect that high-energy particles collide with the CMOS transistor to create an electron-hole pair, and the additional hole is trapped in the gate oxide. TID accumulates for a long time and changes the threshold voltage, Vth, which may increase the leakage current of CMOS transistors used in analog circuits. SEE refers to the effect of generating instantaneous charge when high energy particles collide with the CMOS transistor. SEE temporarily flips the data stored in the digital and memory circuits and changes the voltage of the switched-capacitor circuits. For SEE-tolerant digital circuits, a triple modular redundancy (TMR) technique has been commonly used to reduce errors by SEE, which concept can be also applied for radiation-hardened ADCs. However, TMR requires 3× larger power and silicon area because multiple identical modules are required. Moreover, in case of high-energy radiation environments where each module suffers from higher probability of SEE errors, TMR is not sufficient to tolerate the soft errors. Therefore, CMOS-based ADCs, which operate in radiation environments, should be designed carefully to be robust to both TID and SEE.

Section 2 analyzes the radiation-hardened ADC structures and explains how to design a radiation-hardened ADC with circuit considerations. Section 3 proposes the soft-error-tolerant sample-and-hold (S/H) system using dual-capacitor control. Section 4 shows the measurement results of the proposed radiation-hardened successive-approximation-register (SAR) ADC measured in actual radiation environments and verification results of the proposed dual-capacitor S/H technique with using radiation simulator CAD tools. Finally, Section 5 provides concluding remarks.

## 2. Soft-Error-Tolerant ADC Structure

The ADC type, such as flash ADC, pipelined ADC, sigma-delta ADC, and SAR ADC, can be selected depending on the required sampling rate and resolution. For the sensor system that measures environmental signals, such as electromagnetic field, pressure, and temperature, ADCs usually require a sampling rate above tens of kHz and resolution of 8–10 bit. Therefore, the flash ADC with a limited resolution of up to 6 bit is not suitable for the sensor system. Additionally, the sigma-delta ADC that has a high resolution but operates at a slow sampling rate may not be suitable. As a result of analyzing each ADC in terms of TID tolerance and SEE tolerance, the flash ADC is vulnerable to TID because it uses multiple comparators [9]. The pipelined ADC and the sigma-delta ADC also use multiple comparators and amplifiers, so the performance can be easily degraded by TID [10,11].

The SAR ADC satisfies the sampling rate of more than tens of kHz and 8–10 bit resolution required by the sensor system. In addition, sub-block circuits in the SAR ADC are mostly composed of switched-capacitor circuits and digital circuits, ensuring relatively robust operation against TID [12,13]. While those switched-capacitor and digital circuits can be vulnerable to SEE, the proposed SAR ADC structure was applied to solve this problem.

**Figure 2:**
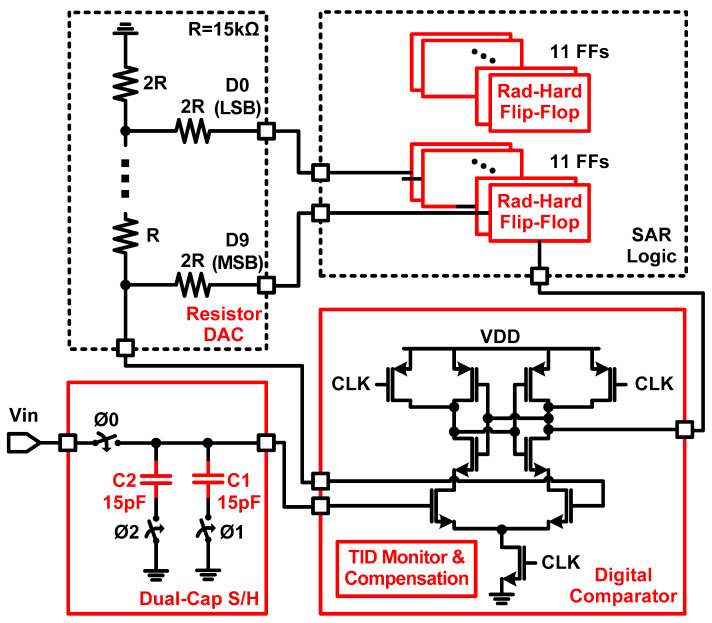
Block diagram of the proposed SAR ADC with dual-capacitor S/H.

Figure 2 shows the proposed SAR ADC structure, which has robust characteristics against TID and SEE. Since a capacitive type digital-to-analog converter (DAC) can be vulnerable to SEE, the proposed SAR ADC utilizes the resistor type DAC to ensure robust operation against SEE. While conventional flip-flops used in SAR logic are also vulnerable to SEE, the total 22 delay-based dual feedback flip-flops, which are required to implement 10-bit resolution, can be used to obtain SEE tolerance [8,14]. In the case of a digital comparator, a TID monitor circuit was added to monitor and compensate for the TID effect inside. Finally, S/H uses the proposed dual-capacitor S/H, which divides one capacitor into two capacitors and controls their operation timing instead of using the conventional S/H with one capacitor, to obtain soft-error tolerance against SEE. While the previous works in [8,15] focused on radiation-hardened flip-flops and an instrumentation amplifier, this paper proposed a soft-error-tolerant SAR ADC with the dual-capacitor S/H and digital comparator.

## 3. Radiation-Hardened Circuit Techniques

### 3.1. Soft-Error-Tolerant Sample-and-Hold (S/H) Control

**Figure 3:**
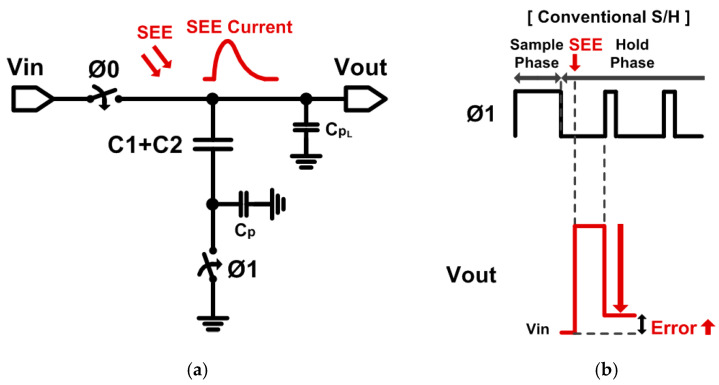
(**a**) Schematic of the conventional single-capacitor S/H. (**b**) Output voltage waveforms.

Figure 3a shows a conventional S/H using a switched-capacitor structure, and the voltage stored in the capacitor, C1+C2, can be changed arbitrarily by SEE. Large capacitors can be used to minimize voltage variations due to SEE, but this increases the circuit area and loading effects. Figure 3b depicts the changes of the sampled output voltage, V_out_, when SEE occurs in the conventional S/H. When SEE occurs after opening the switch by Ø1, the instantaneous charge due to the SEE charges the parasitic capacitor, CPL, increasing the output voltage. Then, Ø1 closes the switch to provide the sampled voltage stored in the capacitor. However, there is still a large error left, which affects the output voltage, due to the unwanted charge by SEE.

**Figure 4:**
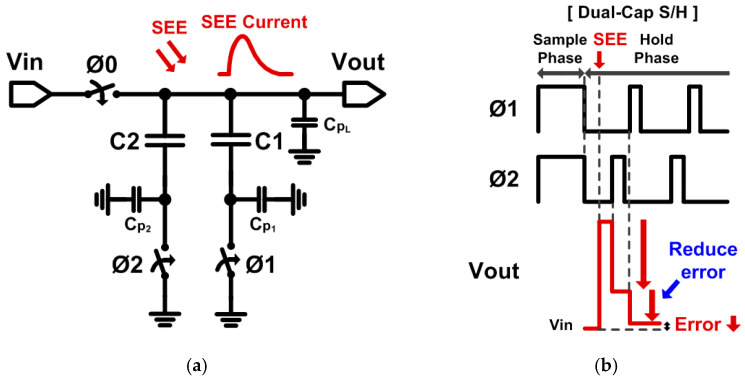
(**a**) Schematic of the proposed dual-capacitor S/H. (**b**) Output voltage waveforms.

Figure 4a shows the proposed dual-capacitor S/H, which has robust characteristics against SEE. The proposed S/H utilizes two capacitors, C1 and C2, and controls the operation timing of each capacitor, while total capacitance is the same as that of the one-capacitor S/H. In the sampling phase, Ø0, Ø1, and Ø2 switches are all turned on, and C1 and C2 are charged together at the same time. When the S/H operates in the hold phase, Ø1 and Ø2 switches are turned off at the same time, and the Ø0 switch is turned off afterward, so that both capacitors become floating. When SEE occur in this hold phase, the C2 capacitor first compensates for errors in V_out_ due to SEE by turning the Ø2 switch on and off just before the digital comparator operates. In other word, the C2 capacitor absorbs some of the errors stored in sampling capacitors. Then, by turning on the Ø1 switch, the C1 capacitor additionally compensate for the errors due to SEE, minimizing the voltage variations in the sampling capacitors. It is noted that SEE during the sampling phase has little effect to V_out_ since the input voltage, V_in_, is still charging the capacitors.

Figure 4b shows the changes of the output voltage when SEE occurs in the proposed dual-capacitor S/H. The charge due to SEE is charged to the parasitic capacitor CPL. When SEE occurs during the hold phase, while the output voltage initially increased by SEE, it lowers primarily because of the C2 capacitor and is then further lowered by the C1 capacitor. As a result, the proposed S/H can be more soft-error tolerant against SEE compared to the conventional S/H. The smaller the size of the parasitic capacitors at the bottom of C1 and C2 capacitors, the larger the effectiveness of the proposed S/H that can be achieved. Considering the typical parasitic capacitance (up to 10–20% of the capacitor) of the sampling capacitors used in ICs, the proposed dual-capacitor S/H still provides an effective solution to sample and maintain the input voltages, suitable for the SAR ADC.

### 3.2. TID-Compensated Digital Comparator

**Figure 5:**
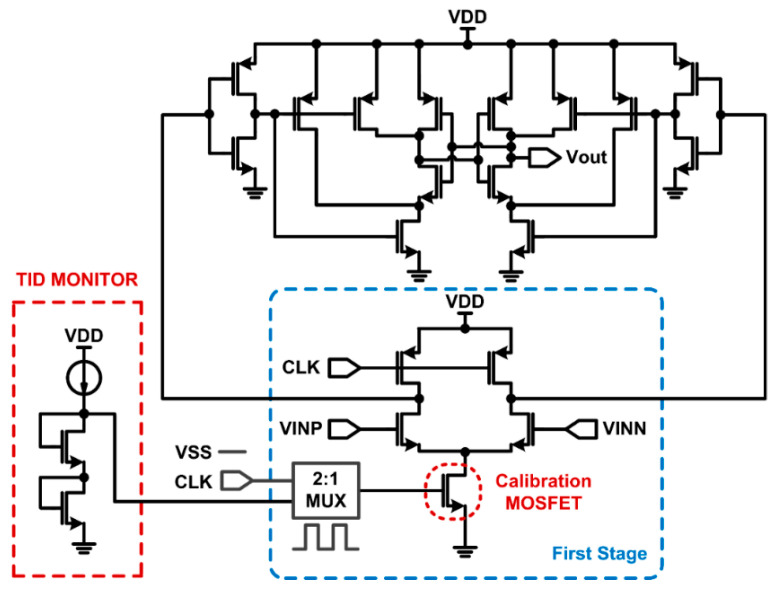
Digital comparator with TID monitoring and compensation functions.

**Figure 6:**
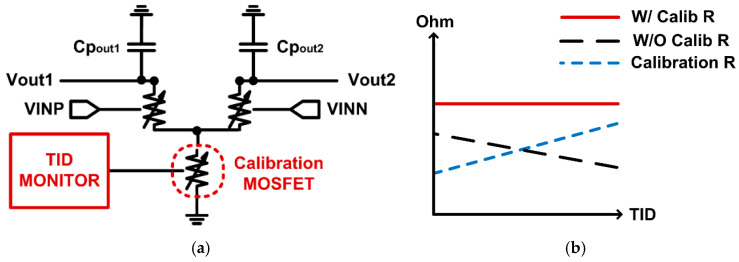
(**a**) A simplified circuit model of the first stage in the digital comparator as variable resistors. (**b**) Resistance at the output node of the first stage in the digital comparator.

Instead of using a TID-sensitive analog comparator, a digital comparator synchronized to the clock signal can be used to obtain robust characteristics against TID, as shown in Figure 5. The comparator consists of two stages, and the PMOS switches connected to the V_DD_ in the first stage turn off when the clock, CLK, increases to start the comparison. In this condition, the first stage can be modeled with resistors as shown in Figure 6a. When the clock is high, the NMOS transistors operating in the triode region can be represented as resistors, and the parameter changes of each NMOS due to TID can be expressed as a variable resistor. CP_out1_ and CP_out2_ represent all parasitic capacitances connected to the nodes of V_out1_ and V_out2_.

The transient response and operation speed in the V_out1_ and V_out2_ nodes can be determined by the resistance and capacitance at each output node. When the modeling resistances are changed by TID, the transient responses are also affected, changing the comparator performance. By monitoring the V_th_ variation of the NMOS transistors and compensating for the resistance value through the calibration NMOS transistor, the comparator can be more robust to TID, maintaining the similar level of operation performance over TID accumulation. Figure 6b shows the concept of the resistance compensation in input transistors of the digital comparator.

## 4. Measurement Results

### 4.1. Test Chip Fabrication and Radiation Test Setup

**Figure 7:**
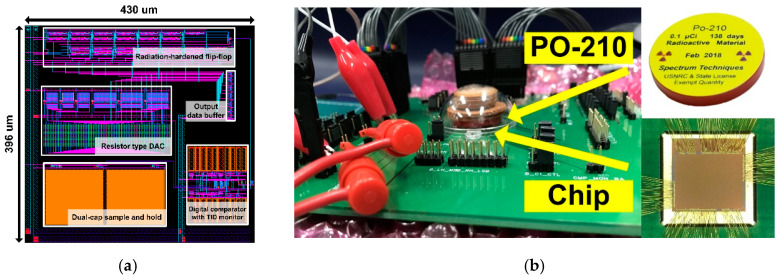
(**a**) The layout floorplan of the SAR ADC. (**b**) Chip radiation test setup with alpha source (PO-210).

Figure 7a shows the layout floorplan of the proposed SAR ADC, which occupies the total silicon area of 0.17 mm^2^ (430 μm × 396 μm). The SAR ADC was fabricated in 65 nm CMOS process, and all sub-blocks are closely located to minimize interconnect parasitic effects. A high-energy alpha source (PO-210) was used to verify the soft-error-tolerant performance of the proposed dual-capacitor S/H against SEE. Figure 7b shows the chip radiation test setup with an alpha source, which was located above the test chip. The test chip was directly wire-bonded on the PCB board without packaging because the package of the chip provides a shielding effect when conducting radiation experiments.

**Figure 8:**
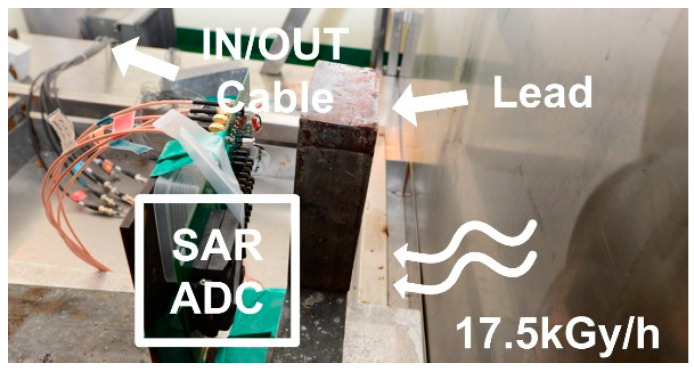
Chip radiation test setup inside the high-level radiation facility.

Additional gamma-ray radiation experiments were also conducted in a high-level gamma-ray irradiation facility of the Advanced Radiation Technology Institute (ARTI). Figure 8 shows the chip test setup in ARTI high-level radiation environments with measurement equipment setting beyond the shielding wall of the radiation facility. In order to verify the radiation-hardened performance of the ADC, the remaining parts on the PCB board except the test chip were shielded with lead.

### 4.2. Test Chip Verification in Radiation Environments

**Figure 9:**
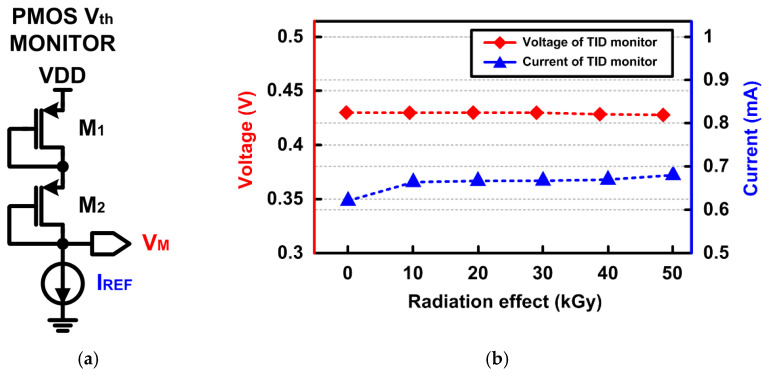
(**a**) Conceptual diagram of the TID-monitoring circuit. (**b**) Measured monitoring voltage against TID up to 50 kGy.

To measure the TID effect, the TID-monitoring circuit of the test chip was exposed to the ARTI high-level radiation environment for 200 min under a high dose rate of 15 kGy/h, resulting in a total ionizing dose of 50 kGy. Figure 9a is a conceptual diagram of the TID-effect monitoring circuit to estimate the TID effect on the transistor threshold voltage, V_th_. Figure 9b shows the monitoring voltage, V_M_, and reference current, I_REF_, according to the TID level, which describes the change in device characteristics, i.e., V_th_. I_REF_ was generated by using the V_th_-insensitive current generator in [15]. It can be observed that the monitoring voltage, which can be defined as V_M_ = V_DD_ − V_SG1_ − V_SG2_, is near constant around 0.43 V, showing that the TID has a small effect on the transistor, up to 50 kGy.

**Figure 10:**
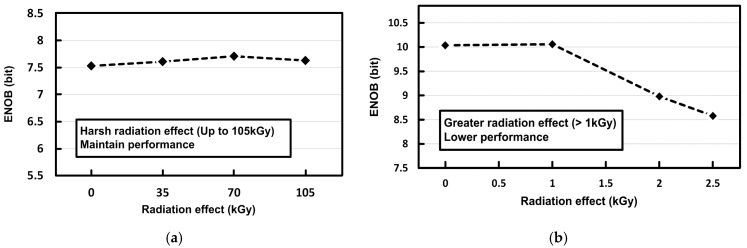
Measured ENOB of (**a**) the proposed radiation-hardened SAR ADC and (**b**) the commercial SAR ADC (ADS7044) against TID effects.

Figure 10a shows the measured effective-number-of-bits (ENOB) of the proposed SAR ADC chip according to the TID levels. The proposed SAR ADC test chip was exposed to the ARTI high-level radiation environments for 6 h under a high dose rate of 17.5 kGy/h, resulting in a total dose of 105 kGy. The proposed SAR ADC maintained its ENOB performance against the high radiation up to 105 kGy thanks to its digital-based architecture in Figure 2, which can be less affected by transistor degradation. The commercial SAR ADC (ADS7044) in Figure 10b suffers from chip performance degradation with radiation effects greater than 1 kGy.

**Figure 11:**
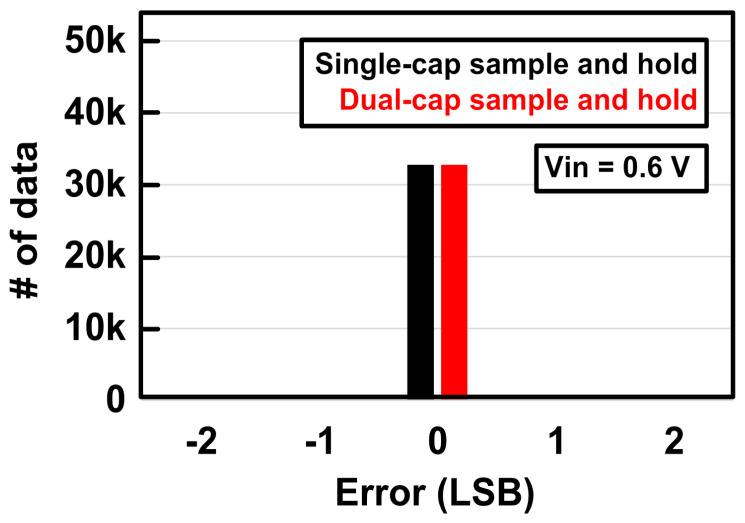
Measurement results of radiation-hardened SAR ADC using alpha source (PO-210).

Figure 11 shows the soft-error-tolerant performance of the proposed dual-capacitor S/H using a high-energy alpha source (PO-210). When the alpha source provides the radiation effects to the SAR ADC chips with both single-capacitor S/H and dual-capacitor S/H, there was no error observed at the SAR ADC outputs. While both S/H circuits do not result in soft errors in the SAR ADC due to the limited radiation test time, the further comparison of the soft-error-tolerant performance between the conventional S/H and the proposed dual-capacitor S/H can be verified through radiation CAD simulations described in Section 4.3.

### 4.3. Verification of the Sample-and-Hold and Digital Comparator

**Figure 12:**
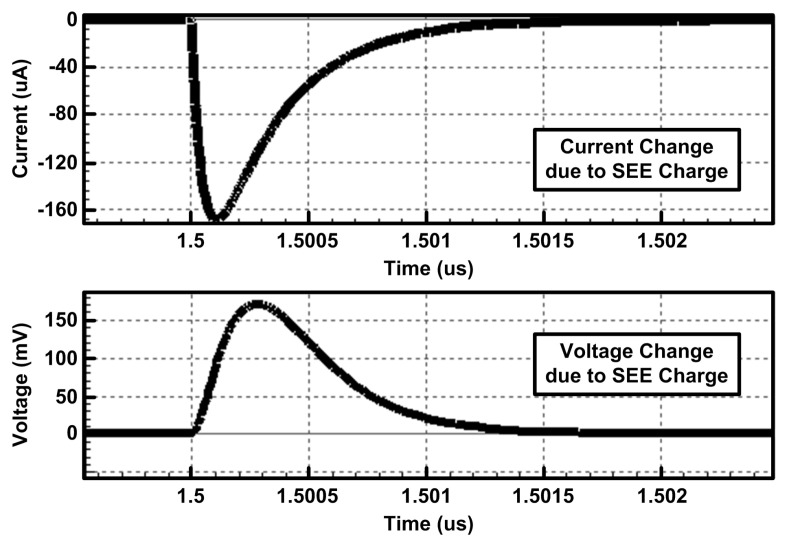
Simulated current and voltage waveforms generated due to SEE [8].

**Figure 13:**
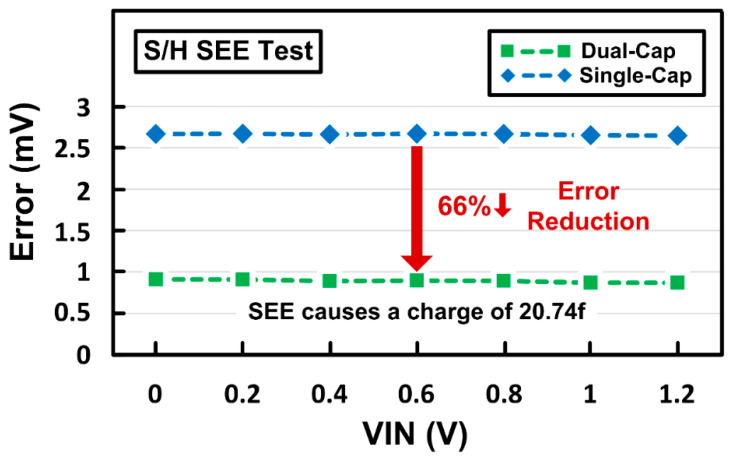
Output errors of single-capacitor and dual-capacitor S/Hs with SEE radiation simulation.

A radiation simulator CAD tool (Silvaco, Santa Clara, CA, USA) and custom-designed simulation setup were used to verify the soft-error-tolerant performance of the proposed dual-cap S/H [16,17]. Figure 12 shows the simulated waveforms of the current and voltage changes due to SEE when provided to the output node of the inverter, which can also be applied to the S/H to affect its sampled voltage values. Figure 13 shows the output error of the S/H due to SEE, which represents the difference between the ideal outputs and the actual outputs affected by SEE when various input voltages are provided. The number of additional charges generated by SEE was set to 20.74 fC. Over a wide range of input voltages, the proposed dual-capacitor S/H has an output error of 0.9 mV, while the conventional single-capacitor S/H suffers from an output error of 2.7 mV, which can degrade the effective resolution of the ADC. The dual-capacitor S/H can reduce the output error by 66% compared to the single-capacitor S/H thanks to its dual-capacitor control for error compensation, leading to robust operation against SEE.

**Figure 14:**
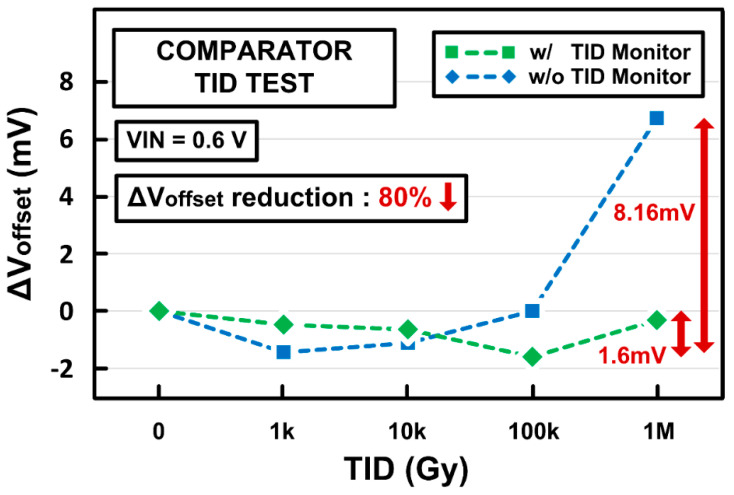
Offset voltage variations of the digital comparator with TID radiation simulation.

Figure 14 shows the offset voltage variations of the proposed digital comparator against TID effects. The initial offset voltage was set intentionally by using the size mismatch between input transistors. While the digital comparator suffers from large offset variation of 8.16 mV with TID levels up to 1 MGy, the proposed TID monitoring and compensation functions can decrease the offset variation to 1.6 mV, resulting in an 80% reduction. Thus, it can be observed that the digital comparator with TID monitoring can maintain a similar offset performance over a wide range of TID levels up to 1 MGy, leading to robust comparator operation against TID.

### 4.4. Performance of the Proposed SAR ADC

**Figure 15:**
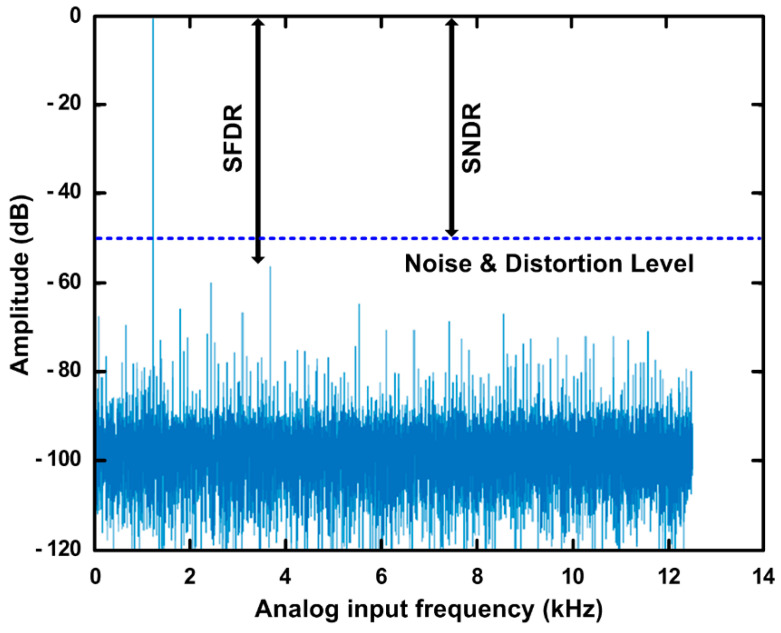
Measured 32,768-point FFT spectrum of the SAR ADC with dual-capacitor S/H at 25 kS/s.

Figure 15 shows the measured 32,768-point FFT spectrum with an input frequency of 1.22 kHz with a 1.2-V supply and a 25 kS/s sampling rate for ADC analysis [18]. The signal-to-noise-distortion-ration (SNDR) and ENOB can be obtained by measuring signal and noise amplitudes. Table 1 summarized the measured performance of the test chip. The measured SNDR and ENOB of the SAR ADC using dual-cap S/H are 49.99 dB and 8.01 bit, respectively. The power consumption of 210 μW roughly results from SAR logic (90 μW), comparator with TID monitoring (90 μW), RDAC (30 μW), and S/H (<1 μW), while buffer amplifiers, references, and I/O buffer inverters are not included. The proposed dual-capacitor S/H improves the soft-error tolerance against SEE, while the SAR ADC performance was not degraded compared to the conventional S/H SAR ADC.

## 5. Conclusions

Integrated circuits used in radiation environments, such as in space, during flights, and in nuclear fusion reactors, suffer from performance degradation due to radiation effects, such as SEE and TID, leading to system malfunctions. Improving the radiation tolerance of the circuit system by utilizing radiation hardening by design (RHBD) along with conventional shielding and process development is highly needed. In this paper, a soft-error-tolerant SAR ADC using a dual-capacitor S/H was proposed, and its radiation-tolerance was measured with a 65-nm CMOS fabricated test chip and radiation simulation tools. The test chip was also verified through actual radiation environments, showing radiation-hardened operation against TID and SEE.

## Figures and Tables

**Table 1:** Performance Summary of the Radiation-Hardened SAR ADC.

Specification (Unit)	Single-Capacitor S/H SAR ADC	Dual-Capacitor S/H SAR ADC
Architecture	SAR	SAR
Technology (nm)	65	65
Supply Voltage (V)	1.2	1.2
Input Range (Vp-p)	1.2	1.2
Sampling Rate (kS/s)	25	25
Resolution (bit)	10	10
SNR (dB)	51.72	51.86
THD (dB)	−55.19	−54.55
SNDR (dB)	50.11	49.99
SFDR (dB)	56.98	56.25
ENOB (bit)	8.03	8.01
Power (μW) *	210	210

* Simulated values for SAR ADC core blocks in Figure 2.

## Data Availability

Not applicable.
